# Photobiomodulation in dentistry: Mechanisms, clinical applications and evidence-based guidelines

**DOI:** 10.6026/973206300214203

**Published:** 2025-11-15

**Authors:** Lana Eltahir, Moustafa Elgendy, Gaya Ali, Abrar Elamin, Sarah Omer, Neerman Khazam

**Affiliations:** 1 Department of Dental Surgery, Lana Eltahir, Sudan; 2 Department of Dental Surgery, Moustafa Elgendy, Egypt; 3 Department of Dental Surgery, Gaya Ali, Sudan; 4 Department of Dental Surgery, Abrar Elamin, Sudan; 5 Department of Dental Surgery, Sarah Omer, Libya; 6 Department of Dental Surgery, Neerman Khazam, Libya

**Keywords:** Photobiomodulation, low-level laser therapy, oral mucositis, temporomandibular disorders, periodontitis, endodontics, dental implants, orthodontics, laser therapy, PBM safety, pain management, tissue regeneration

## Abstract

Photobiomodulation (PBM), formerly known as low-level laser therapy (LLLT), utilizes red and near-infrared light to activate mitochondrial chromophores, thereby enhancing cellular activity, modulating inflammation and promoting tissue repair. Therefore, it is of interest to the mechanistic pathways of PBM, including mitochondrial stimulation, cytokine regulation and growth factor induction. Clinical evidence supports its efficacy in managing oral mucositis, temporomandibular disorders, periodontitis, postoperative endodontic pain, implant stability and orthodontic treatment outcomes.

## Background:

Photobiomodulation (PBM) represents a significant advancement in dental therapeutics, offering non-invasive modulation of tissue healing and pain through targeted light energy [1]. PBM utilizes low-level red and NIR light (600-1000 nm), which is absorbed by mitochondrial chromophores, particularly cytochrome C oxidase, triggering a cascade of photochemical reactions [1]. These include increased adenosine triphosphate (ATP) production, enhanced cell signaling via reactive oxygen species (ROS) and promotion of tissue regeneration without thermal damage [2]. PBM is a non-invasive method that contributes to pain relief and reduces inflammation, while also enhancing healing and tissue repair processes [3]. Therefore, it is of interest to describe an integrative synthesis of PBM's biological mechanisms, clinical efficacy, treatment guidelines and safety considerations, aiming to bridge the gap between bench side evidence and clinical implementation.

## Mechanisms of action:

The therapeutic benefits of PBM are mediated through complex, yet well-characterized, cellular and molecular pathways:

## Mitochondrial stimulation:

PBM enhances activity within the mitochondrial respiratory chain, particularly complex IV (cytochrome c oxidase), leading to increased ATP production. This energy supports various cellular processes, including proliferation, migration and survival [1].

## Reactive oxygen species modulation:

At therapeutic levels, PBM induces transient, controlled increases in ROS. These function as intracellular messengers that promote anti-inflammatory signaling, angiogenesis and wound repair [2].

## Gene expression and cytokine regulation:

PBM modulates transcription factors such as NF-κB and AP-1, promoting the expression of anti-inflammatory cytokines and genes involved in tissue regeneration [4].

## Growth factor release:

Exposure to PBM stimulates growth factor-beta (TGF-β), which enhances fibroblast proliferation, collagen deposition and vascularization [5].

## Clinical applications in dentistry

## Oral mucositis (OM)

PBM is most strongly validated in the prevention and treatment of OM in oncology patients. A 2025 randomized clinical trial demonstrated a dramatic reduction in OM incidence (13.3% in PBM group vs. 93.3% in control) with the use of 650 nm intraoral and 980 nm extraoral diode lasers [6]. Pediatric patients receiving PBM also experienced shorter hospital stays and improved oral intake [7].

## Temporomandibular disorders (TMD):

Meta-analyses of over 40 randomized controlled trials confirm PBM's efficacy in reducing myofascial pain, improving jaw mobility and alleviating joint clicking in TMD patients. PBM modulates nociceptive pathways and reduces inflammatory cytokine activity in the temporomandibular joint (TMJ) region [8].

## Periodontal therapy:

As an adjunct to scaling and root planing (SRP), PBM has been shown to enhance gingival healing, with increased fibroblast activity and reduced apoptosis in periodontal ligament stem cells [9]. A randomized clinical trial reported improved clinical attachment gain and reduced bleeding on probing with adjunctive PBM at 980 nm [10].

## Endodontics:

PBM significantly reduces postoperative endodontic pain. In comparative studies, patients treated with PBM required fewer NSAIDs and reported lower pain scores at 12, 24 and 48 hours post-treatment. Mechanistically, PBM desensitizes peripheral nociceptors and enhances endogenous opioid release, including β-endorphins [11].

## Implantology:

PBM facilitates osseointegration and stability of the implant by stimulating osteoblast proliferation, promoting bone formation and mineralization and angiogenesis. A 2024 systematic review found improved implant stability indices at both 2 weeks and 12 months following surgery in PBM-treated groups [12].

## Orthodontics:

PBM accelerates tooth movement and reduces discomfort during orthodontic treatment and the range from 730 to 830 nm has been considered the most effective. Studies show lower pain perception with the use of wavelength 830 nm while treating pain inherent to tooth movement caused by orthodontic devices [13, 14]. Continued advancements might pave the way for non-invasive or minimally invasive orthodontic treatments. This approach may be particularly beneficial for patients who prefer minimally invasive modalities over conventional orthodontic appliances, while still aiming to achieve enhanced efficiency in tooth movement [15].

## Additional indications:

[1] Dentin Hypersensitivity: PBM modulates odontoblast activity and reduces neural excitability [16].

[2] Post-Surgical Healing: Accelerated reepithelialization, lower presence of inflammatory cells, a more mature epithelium and myofibroblasts incorporated in a network of fibers parallel to the basal membrane following extractions has been seen [17].

[3] Dental Bleaching: Violet LED PBM improves whitening results and minimizes sensitivity when used adjunctively [18].

[4] Reduction in inflammation, control of pain, and improved oral health-related quality of life in various surgical procedures, including sinus lift surgery and dental implant therapy, have been seen. The use of LLLT in conjunction with growth factors has shown promising outcomes in managing complications such as dry sockets and promoting bone osseointegration around dental implants [19].

## Treatment parameters and clinical guidelines:

Effective PBM treatment depends on the precise calibration of key parameters:

[1] Wavelength: 600-1000 nm, selected based on target tissue depth

[2] Energy Density (Fluence): 1-10 J/cm^2^ for most dental applications

[3] Power Density (Irradiance): Typically <500 mW/cm^2^

[4] Application Duration: 30-90 seconds per point

[5] Frequency: 2-3 sessions per week, tailored to clinical need

[6] Guidelines from WALT emphasize the importance of standardized dosimetry to ensure reproducibility and therapeutic efficacy. Variability in PBM parameters across studies has led to inconsistencies in clinical outcomes, highlighting the necessity for adherence to established protocols [20].

## Safety considerations:

PBM has an excellent safety profile when used appropriately. Common precautions include:

[1] Mandatory eye protection: Patients and clinicians must wear goggles to avoid retinal exposure [21].

[2] Thermal safety: Excessive tissue destruction by direct ablation and thermal side effects, including thermal injury to the root surface, gingival tissue and pulp, and bone tissue can occur [22].

## Contraindications include:

[1] In cases of hyperthyroidism.

[2] Application during early pregnancy

[3] Direct exposure to apparent tumor mass [21].

## Conclusion:

Photobiomodulation is a safe, non-invasive and efficacious modality that addresses pain, inflammation and healing across diverse dental specialties. Robust clinical data support its utility in oral mucositis, TMD, periodontal therapy, endodontics, implantology and orthodontics. The standardization of treatment parameters and safety adherence is critical for consistent results. As dental technologies evolve, PBM is expected to become an integral component of personalized and evidence-based dental therapeutics.
